# Ventral-stream-like shape representation: from pixel intensity values to trainable object-selective COSFIRE models

**DOI:** 10.3389/fncom.2014.00080

**Published:** 2014-07-30

**Authors:** George Azzopardi, Nicolai Petkov

**Affiliations:** Intelligent Systems, Johann Bernoulli Institute for Mathematics and Computer Science, University of GroningenGroningen, Netherlands

**Keywords:** hierarchical representation, object recognition, shape, ventral stream, vision and scene understanding, robotics, handwriting analysis

## Abstract

The remarkable abilities of the primate visual system have inspired the construction of computational models of some visual neurons. We propose a trainable hierarchical object recognition model, which we call *S*-COSFIRE (*S* stands for *Shape* and COSFIRE stands for *Combination Of Shifted FIlter REsponses*) and use it to localize and recognize objects of interests embedded in complex scenes. It is inspired by the visual processing in the ventral stream (V1/V2 → V4 → TEO). Recognition and localization of objects embedded in complex scenes is important for many computer vision applications. Most existing methods require prior segmentation of the objects from the background which on its turn requires recognition. An *S*-COSFIRE filter is automatically configured to be selective for an arrangement of contour-based features that belong to a prototype shape specified by an example. The configuration comprises selecting relevant vertex detectors and determining certain blur and shift parameters. The response is computed as the weighted geometric mean of the blurred and shifted responses of the selected vertex detectors. *S*-COSFIRE filters share similar properties with some neurons in inferotemporal cortex, which provided inspiration for this work. We demonstrate the effectiveness of *S*-COSFIRE filters in two applications: letter and keyword spotting in handwritten manuscripts and object spotting in complex scenes for the computer vision system of a domestic robot. *S*-COSFIRE filters are effective to recognize and localize (deformable) objects in images of complex scenes without requiring prior segmentation. They are versatile trainable shape detectors, conceptually simple and easy to implement. The presented hierarchical shape representation contributes to a better understanding of the brain and to more robust computer vision algorithms.

## 1. Introduction

Shape is perceptually the most important visual characteristic of an object. Although there is no formal definition—as with most perceptual related concepts—it is understood that the two-dimensional shape of an object is characterized by the relative spatial positions of a collection of contour-based features.

**Figure 1:**
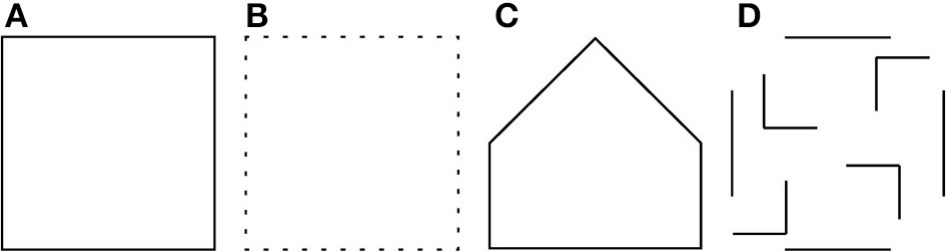
**(A)** A prototype shape. **(B)** A test pattern that has only 25% similarity (computed by template matching) to the prototype is perceptually more similar to the prototype than the polygon in **(C)** and the set of contour parts in **(D)**, both of which have 50% similarity (computed by template matching) to the prototype.

Let us consider, for instance, the square in Figure 1A, which we refer to as a reference or prototype object. From the point of view of visual perception the incomplete object in Figure 1B is very similar to the prototype even though it is composed of only 25% of the contour pixels of the reference object. On the contrary, the closed polygon in Figure 1C, which has the bottom half equivalent to that of the prototype is perceptually less similar to it. Furthermore, there is little perceptual similarity between the prototype and its scrambled contour parts shown in Figure 1D.

**Figure 2:**
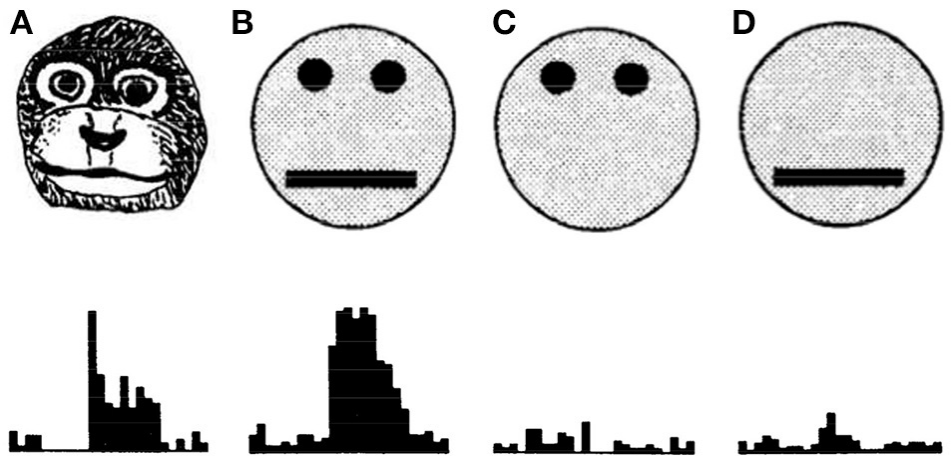
**(A–D)** A set of stimuli used in an electrophysiological study Kobatake and Tanaka (1994) to test the selectivity of a neuron in inferotemporal cortex. **(Bottom)** The activity of the concerned neuron for the corresponding stimuli. The neuron gives high response only when the stimulus contains a detailed or simplified representation of the face boundary that surrounds a pair of eyes on top of a mouth. If any of these features is missing, the neuron gives negligible response.

As a matter of fact, there is neurophysiological evidence that objects, such as faces, are recognized by detecting certain features that are spatially arranged in a certain way (Kobatake and Tanaka, 1994). By means of single-cell recordings in adult monkeys it was, for instance, found that a neuron in inferotemporal cortex gives similar responses for the two images shown in Figures 2A,B. The icon presented in Figure 2B is a simplified version of the monkey's face shown in Figure 2A. It only consists of a circle that surrounds a horizontally-aligned pair of spots on top of a horizontal bar. Removing one of these features, Figures 2C,D, causes the concerned cell to give very small response.

Another neurophysiological study (Brincat and Connor, 2004) reveals that some neurons in inferotemporal cortex integrate information about the curvatures, orientations, and positions of multiple (typically 2–4) simple contour elements, such as angles or curved contour segments. In that study the authors argue that their findings are in line with other studies that support parts-based shape representation theories (Marr and Nishihara, 1978; Riesenhuber and Poggio, 1999; Mel and Fiser, 2000; Edelman and Intrator, 2003), and suggest that non-linear integration in the inferotemporal cortex might help to extend sparseness of shape representation along the ventral stream.

Tsotsos (1990) showed that hierarchical architectures are more appropriate for object detection in contrast to unbounded visual search which is known to be NP-complete. This has led to the proposal of a number of hierarchical models (Mel and Fiser, 2000; Scalzo and Piater, 2005; DiCarlo and Cox, 2007; Rodríguez-Sánchez and Tsotsos, 2012). Existing approaches that consider the spatial relationship of features include the so-called standard model (Serre et al., 2007), some probabilistic techniques, such as the generative constellation model (Fergus et al., 2003; Fei-Fei et al., 2007) and a hierarchical model of object categories (Fidler and Leonardis, 2007; Fidler et al., 2008). These approaches rely on summation of the responses of elementary feature detectors and may find the images in Figures 1C,D quite similar to the prototype in Figure 1A. For instance, such a technique may consider a circle with a horizontal line within it as a face even though the representations of the eyes are missing, Figures 2C,D.

We introduce a hierarchical object detection technique which is motivated by the shape selectivity of some neurons in inferotemporal cortex. The principal idea is to construct a shape-selective filter that combines the responses of some simpler filters that detect some partial features of the concerned shape in specific positions that are characteristic of that shape. We call this approach to the construction of filters Combination Of Shifted Filter REsponses (COSFIRE). We successfully applied this approach to the construction of line and edge detectors (Azzopardi and Petkov, 2012; Azzopardi et al., 2014) and simple contour-related features, such as vascular bifurcations (Azzopardi and Petkov, 2013b). In Azzopardi and Petkov (2013b) we demonstrated how the collective responses of multiple COSFIRE filters to segmented patterns, such as handwritten digits, can be used to form a shape descriptor with high discrimination ability. That descriptor, however, does not take into account the relative spatial arrangement of the concerned features. Similar to other shape descriptors (Belongie et al., 2002; Grigorescu and Petkov, 2003; Ghosh and Petkov, 2005; Latecki et al., 2005; Lauer et al., 2007; Ling and Jacobs, 2007; Goh, 2008; Almazan et al., 2012) that approach works well with segmented objects, but it is not effective for the detection of objects embedded in complex scenes. In order to distinguish the two types of filter, we refer to the composite shape-selective filter that we propose in this paper as *S*-COSFIRE and to the filter proposed in Azzopardi and Petkov (2013b) as *V*-COSFIRE (*S* and *V* stand for shape and vertex, respectively).

There are three aspects in which the *S*-COSFIRE filters that we propose differ from other hierarchical models that also consider the spatial geometric arrangement of parts. *First*, our model is implemented in a filter that gives a scalar response (between 0 and 1) for each position in the image. The higher the value the more similar the shape around the concerned location is to the prototype shape. An *S*-COSFIRE filter can be thought of a model of a shape-selective neuron in inferotemporal cortex of the type studied in Kobatake and Tanaka (1994); Brincat and Connor (2004), which fires only when a specific arrangement of contour-based features is present in its receptive field. It addresses object recognition and localization as a joint problem, which is in line with how Marr (1982) defined the sense of seeing: “… to know what is where by looking.” In contrast, the other methods referred to above use multiple prototypes and consider several responses from different feature detectors to form a mixture of probability distributions or a vector of responses. For these methods, the geometrical spatial arrangement of the concerned prototype defining parts is achieved by training a supervised classifier and subsequently the similarity between a test pattern and a prototype is computed by a distance metric. Moreover, they suffer from insufficient robustness to localization because they treat this matter at a region level (sliding window) rather than at a pixel level.

*Second*, since the omission of an object part can radically change shape perception, we regard every feature (and its relative position) that forms part of a prototype shape as essential. This aspect is implemented as an AND-type operation of an *S*-COSFIRE filter. It is in contrast to other models that rely on summation, and therefore achieve a response even when any of the prototype-defining features is missing. These models may thus match objects that are perceptually different.

*Third*, while the *S*-COSFIRE approach that we present achieves invariance to rotation, scaling, and reflection by simply manipulating some model parameters, the other techniques can only achieve invariance to such geometric transformations by extending the training set with example objects that are rotated, scaled and/or reflected versions of a prototype.

The rest of the paper is organized as follows: in section 2 we present the proposed hierarchical *S*-COSFIRE model. In section 3, we demonstrate its effectiveness in two applications: keyword spotting in handwritten manuscripts and vision for a home tidying pickup robot. Section 4 contains a discussion on the properties of the *S*-COSFIRE filters and finally we draw conclusions in section 5.

## 2. Methods

**Figure 3:**
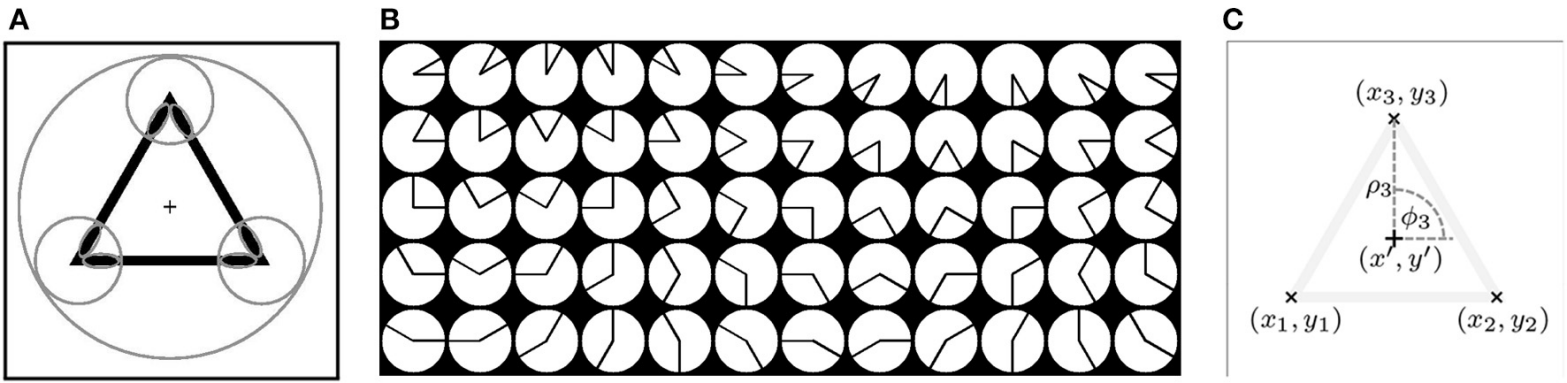
**(A)** The triangle is the prototype shape of interest and the “+” marker indicates the center of the user-specified large circle. The small circles indicate the supports of three vertex detectors that are identified as relevant for the concerned prototype shape. The small ellipses represent the supports of line detectors that are selective for the contour parts of the corresponding vertices. **(B)** A data set of 60 synthetic vertices, *f*_1_, …, *f*_60_ (left-to-right, top-to-bottom). A *V*-COSFIRE filter *V*_*f*_*k*__ is selective for a vertex *f*_*k*_. **(C)** Configuration of an *S*-COSFIRE filter. The “×” markers indicate the locations, (*x*_1_, *y*_1_), (*x*_2_, *y*_2_), (*x*_3_, *y*_3_), where the corresponding three *V*-COSFIRE filters, *V*_*f*_13__, *V*_*f*_17__, *V*_*f*_21__, achieve the maximum responses. These locations correspond to the three vertices of the prototype shape, which is rendered here with low contrast. The Cartesian coordinates of each point (*x*_*i*_, *y*_*i*_) are converted into the polar coordinates (ρ_*i*_, ϕ_*i*_) with respect to the given point of interest (*x*′, *y*′), indicated by the “+” marker.

The following example illustrates the main idea of the proposed method. We consider the triangle, shown in Figure 3A, as a shape of interest and we call it *prototype*. We use this prototype to automatically configure an *S*-COSFIRE filter that will respond to shapes that are identical with or similar to this prototype.

A shape-selective *S*-COSFIRE filter takes input from simpler filters; here filters that are selective for vertices. We use vertex-selective COSFIRE filters of the type proposed in Azzopardi and Petkov (2013b) to detect the vertices of the prototype shape. Such a filter, which we refer to it as *V*-COSFIRE, combines the responses of line detectors, the areas of support of which are indicated by the small ellipses in Figure 3A.

The response of an *S*-COSFIRE filter is computed by combining the responses of the concerned *V*-COSFIRE filters in the centers of the corresponding circles by weighted geometric mean. The preferred orientations and the preferred apertures of these filters together with the locations at which we take their responses are determined by analysing the responses of a set of *V*-COSFIRE filters to the prototype shape. Consequently, the *S*-COSFIRE filter will be selective for the given spatial arrangement of vertices of specific orientations and apertures. Taking the responses of *V*-COSFIRE filters at different locations around a point can be implemented by shifting the responses appropriately before using them for the pixel-wise evaluation of a multivariate function which gives the *S*-COSFIRE filter output.

### 2.1. Detection of vertex features by *V*-COSFIRE filters

We denote by *r*_*V*_*f*_*i*___(*x, y*) the response of a *V*-COSFIRE filter *V*_*f*_*i*__ that is selective for a vertex *f*_*i*_. We threshold these responses at a given fraction *t*_1_ (0 ≤ *t*_1_ ≤ 1) of the maximum response across all image coordinates (*x, y*) and denote these thresholded responses by |*r*_*V*_*f*_*i*___(*x, y*)|_*t*_1__. We use the publicly available Matlab implementation^1^ of *V*-COSFIRE filters. Such a filter uses as input the responses of given channels of a bank^2^ of Gabor filters. For further technical details about the properties of *V*-COSFIRE filters we refer to Azzopardi and Petkov (2013b).

We use a bank of *V*-COSFIRE filters that are selective for vertices of different orientations (in intervals of π/6 radians) and different apertures (in intervals of π/6 radians), Figure 3B. For the considered prototype the strongest responses are obtained by three *V*-COSFIRE filters that are selective for vertices of the types *f*_13_, *f*_17_, and *f*_21_, shown in Figure 3B. The corresponding locations, (*x*_1_, *y*_1_), (*x*_2_, *y*_2_), (*x*_3_, *y*_3_), at which they obtain the maximum responses are indicated in Figure 3C.

### 2.2. Configuration of an *S*-COSFIRE filter

An *S*-COSFIRE filter uses as input the responses of selected *V*-COSFIRE filters *V*_*f*_*j*_*i*___, *i* = 1 … *n*, each selective for some vertex *f*_*j*_*i*__, around a certain position (ρ_*i*_, ϕ_*i*_) with respect to the center of the *S*-COSFIRE filter. A 3-tuple (*V*_*f*_*j*_*i*___, ρ_*i*_, ϕ_*i*_) that consists of a *V*-COSFIRE filter specification *V*_*f*_*j*_*i*___ and two scalar values (ρ_*i*_, ϕ_*i*_) characterizes the properties of a vertex that is present in the given prototype shape: *V*_*f*_*j*_*i*___ represents a *V*-COSFIRE filter that is selective for a vertex *f*_*j*_*i*__ and (ρ_*i*_, ϕ_*i*_) are the polar coordinates of the location at which its response is taken with respect to the center of the *S*-COSFIRE filter. In the following we explain how we obtain the parameter values of such vertices around a given point of interest.

For each location in the input image of the prototype shape we take the maximum value of all responses achieved by the bank of *V*-COSFIRE filters mentioned above. The positions that have values greater than those of their corresponding 8-neighbors are chosen as the points that have local maximum responses. For each such point (*x*_*i*_, *y*_*i*_) we determine the polar coordinates (ρ_*i*_, ϕ_*i*_) with respect to the center of the *S*-COSFIRE filter, Figure 3C. Then we determine the *V*-COSFIRE filters, the responses of which are greater than a fraction *t*_2_ = 0.75 of the maximum response *r*_*V*_*f*_*i*___(*x, y*) for all *i* ∈ {1, … *n*_*f*_} where *n*_*f*_ is the number of *V*-COSFIRE filters used across all locations in the input image. Thus, multiple *V*-COSFIRE filters can be significantly activated for the same location (ρ_*i*_, ϕ_*i*_). The selected points characterize the dominant vertices in the given prototype shape of interest.

We denote by *S*_S_ = {(*V*_*f*_*j*_*i*___, ρ_*i*_, ϕ_*i*_) | *i* = 1 … *n*_*f*_} the set of parameter value combinations, which describes the properties and locations of a number of vertices. The subscript S stands for the prototype shape of interest. Every tuple in set *S*_S_ specifies the parameters of some vertex in prototype S. For the prototype shape of interest in Figure 3A, the selection method described above results in three vertices with parameter values specified by the tuples in the following set: *S*_S_ = {(*V*_*f*_*j*_1_ = 21__, ρ_1_ = 50, ϕ_1_ = π/2), (*V*_*f*_*j*_2_ = 13__, ρ_2_ = 50, ϕ_2_ = 7π/6), (*V*_*f*_*j*_3_ = 17__, ρ_3_ = 50, ϕ_3_ = 5π/3)}.

### 2.3. Blurring and shifting *V*-COSFIRE responses

The above configuration results in an *S*-COSFIRE filter that is selective for a preferred spatial arrangement of three vertices forming an equilateral triangle. Next, we use the responses of the *V*-COSFIRE filters that are selective for the corresponding vertices to compute the output of the *S*-COSFIRE filter as follows.

First, we *blur* the responses of the *V*-COSFIRE filters in order to allow for some tolerance in the position of the respective vertices. This increases the generalization ability of the *S*-COSFIRE filter under construction. We define the blurring operation as the computation of maximum value of the weighted thresholded responses of a *V*-COSFIRE filter. For weighting we use a Gaussian function *G*_σ_(*x, y*), the standard deviation σ of which is a linear function of the distance ρ from the center of the *S*-COSFIRE filter: σ = σ_0_ + αρ where σ_0_ and α are constants. The choice of this linear function is inspired by the visual system of the brain for which we provide more detail in section 4. For α > 0, which we use, the tolerance to the position of the respective vertices increases with an increasing distance ρ from the support center of the concerned *S*-COSFIRE filter.

Second, we *shift* the blurred responses of each *V*-COSFIRE filter by a distance ρ_*i*_ in the direction opposite to ϕ_*i*_. With this shifting the concerned *V*-COSFIRE filter responses, which are located at different positions (ρ_*i*_, ϕ_*i*_) meet at the support center of the *S*-COSFIRE filter. The output of the *S*-COSFIRE filter can then be evaluated as a pixel-wise multivariate function of the shifted and blurred responses of *V*-COSFIRE filter responses. In polar coordinates, the shift vector is specified by (ρ_*i*_, ϕ_*i*_ + π), and in Cartesian coordinates, it is (Δ*x*_*i*_, Δ*y*_*i*_) where Δ*x*_*i*_ = −ρ_*i*_ cos ϕ_*i*_, and Δ*y*_*i*_ = −ρ_*i*_ sin ϕ_*i*_. We denote by *s*_*V*_*f*_*j*_*i*____, ρ_*i*_, ϕ_*i*_(*x, y*), the blurred and shifted thresholded response of a *V*-COSFIRE filter that is specified by the *i*-th tuple (*V*_*f*_*j*_*i*___, ρ_*i*_, ϕ_*i*_) in the set *S*_S_:

(1) $$\begin{array}{l} s_{V_{f_{j_{i}}},\rho_{i},\phi_{i}}(x,y)\overset{\text{def}}{=}\underset{x^{\prime },y^{\prime }}{\max}\left\{{\left|r_{V_{f_{j_{i}}}}(x\text{​}-\text{​}x^{\prime }\text{​​}-\text{​​}\Delta x_{i},y\text{​​}-\text{​​}y^{\prime }\text{​​}-\text{​​}\Delta y_{i})\right|}_{t_{1}}G_{\sigma }(x^{\prime },y^{\prime })\right\},\\ \text{ where}-3\sigma \leq x^{\prime },y^{\prime }\leq 3\sigma \end{array}$$

**Figure 4:**
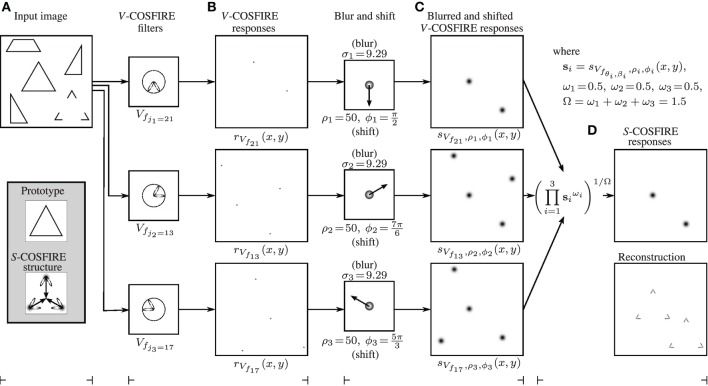
**(A)** Input image (of size 512 × 512 pixels). The enframed inlay images show (top) the enlarged prototype shape of interest, which is identical to the equilateral triangle in the input image and (bottom) the structure of the *S*-COSFIRE filter that is configured by this prototype. The ellipses illustrate the wavelengths and orientations of the Gabor filters that are used by the *V*-COSFIRE filters, and the dark blobs are intensity maps for blurring (Gaussian) functions. The blurred responses are then shifted by the corresponding vectors. **(B)** The *V*-COSFIRE filters that are automatically identified from the prototype shape and the corresponding response images to the input image. **(C)** We then blur (here we use σ_0_ = 0.1 and α = 0.0853 to compute σ_*i*_) the thresholded (here at *t*_1_ = 0) response |*r*_*V*_*f*_*i*___(*x, y*)|_*t*_1__ of each concerned *V*-COSFIRE filter and subsequently shift the resulting blurred response images by corresponding polar-coordinate vectors (ρ_*i*_, ϕ_*i*_ + π). **(D)** We use weighted geometric mean (here σ′ = 91.44) of all the blurred and shifted *V*-COSFIRE filters to compute (top) the output of the *S*-COSFIRE filter and show (bottom) the reconstruction of the detected features. The reconstruction is achieved by superimposing the Gabor filter responses that give input to the *S*-COSFIRE filter. The two local maxima in the output of the *S*-COSFIRE filter correspond to the triangle and to the perceived one in the input image. For better clarity we use inverted gray-level rendering to show the images in the right of the columns **(B–D)**.

Figure 4 illustrates the blurring and shifting operations for this *S*-COSFIRE filter, applied to the image shown in Figure 3A.

We define the response *r*_*S*_S__ (*x, y*) of an *S*-COSFIRE filter as the weighted geometric mean of the blurred and shifted thresholded responses of the selected *V*-COSFIRE filters *s*_*V*_*f*_*j*_*i*____, ρ_*i*_, ϕ_*i*_ (*x, y*):

(2) $$\begin{array}{l} r_{S_{\text{S}}}(x,y)\overset{\text{def}}{=}{\left|{\left(\prod_{i\;=\;1}^{\left|S_{\text{S}}\right|}{\left(s_{V_{f_{j_{i}}},\rho_{i},\phi_{i}}(x,y)\right)}^{\omega_{i}}\right)}^{1/\sum_{i=1}^{\left|S_{\text{S}}\right|}\omega_{i}}\right|}_{t_{3}},\\ \;\omega_{i}=\exp^{-\frac{\rho_{i}^{2}}{2{\sigma^{\prime }}^{2}}},\;0\leq t_{3}\leq 1 \end{array}$$

where |.|_*t*_3__ stands for thresholding the response at a fraction *t*_3_ of its maximum across all image coordinates (*x, y*). For 1/σ′ = 0, the computation of the *S*-COSFIRE filter is equivalent to the standard geometric mean, where the *s*-quantities have the same contribution. Otherwise, for 1/σ′>0, the input contribution of *s*-quantities decreases with an increasing value of the corresponding parameter ρ. In our experiments we use a value of the standard deviation σ′ that is computed as a function of the maximum value of the given set of ρ values: σ′ = (− ρ_max_^2^/2 ln 0.5)^1/2^, where ρ_max_ = max_*i*∈{1…|*S*_S_|}_{ρ_*i*_}. We make this choice in order to achieve a maximum value ω = 1 of the weights in the center (for ρ = 0), and a minimum value ω = 0.5 in the periphery (for ρ = ρ_max_).

Figure 4D shows the output of an *S*-COSFIRE filter which is defined as the weighted geometric mean of three blurred and shifted response images obtained by the three concerned *V*-COSFIRE filters. Note that this filter responds in the middle of a spatial arrangement of three vertices that is identical with or similar to that of the prototype shape S, which was used for the configuration of the *S*-COSFIRE filter. In this example, the *S*-COSFIRE filter reacts strongly in a given point that is surrounded by three vertices each having an aperture of π/3 radians: one northward-pointing, another one south-west-pointing and a south-east-pointing vertex to the north, south-west, and south-east of that point, respectively. Besides the complete triangle that was used for configuration, the concerned filter also detects the Kanizsa-type illusory triangle. This is in line with neurophysiological and psychophysical evidence, in that the visual system is capable of detecting a shape with illusory contours, based on its visible salient parts. A thorough review of this phenomenon is provided in Roelfsema (2006).

### 2.4. Tolerance to geometric transformations

The proposed *S*-COSFIRE filters are tolerant to rotations, scales and reflections. Similar to a *V*-COSFIRE filter, such a tolerance is achieved by manipulating the values of some parameters rather than by configuring separate filters by rotated, scaled, and reflected versions of the prototype shape of interest.

### 2.5. Tolerance to rotation

Using the set *S*_S_ that defines the concerned *S*-COSFIRE filter, we form a new set ℜ_ψ_(*S*_S_) that defines a new filter, which is selective for a version of the prototype shape S that is rotated by an angle ψ:

(3) $${ℜ}_{\psi }(S_{\text{S}})\overset{\text{def}}{=}\left\{({ℜ}_{\psi }(V_{f_{j_{i}}}),\rho_{i},\phi_{i}\text{​}+\text{​}\psi )\;|\;\forall \;(V_{f_{j_{i}}},\rho_{i},\phi_{i})\text{ ​}\in \text{ ​}S_{\text{S}}\right\}$$

For each tuple (*V*_*f*_*j*_*i*___, ρ_*i*_, ϕ_*i*_) in the original filter *S*_S_ that describes a certain vertex of the prototype shape, we provide a counterpart tuple (ℜ_ψ_(*V*_*f*_*j*_*i*___), ρ_*i*_, ϕ_*i*_ + ψ) in the new set ℜ_ψ_(*S*_S_). The set ℜ_ψ_(*V*_*f*_*j*_*i*___) defines^3^ a *V*-COSFIRE filter that is selective for vertex *f*_*j*_*i*__ that is also rotated by an angle ψ. The orientation of the concerned vertex and its polar angle position ϕ_*i*_ with respect to the support center of the *S*-COSFIRE filter are off-set by an angle ψ relative to the values of the corresponding parameters of the original vertex.

A rotation-invariant response is achieved by taking the maximum value of the responses of filters that are obtained with different values of the parameter ψ:

(4) $${\hat{r}}_{S_{\text{S}}}(x,y)\overset{\text{def}}{=}\underset{\psi \;\in \;\Psi }{\max}\left\{r_{{ℜ}_{\psi }(S_{\text{S}})}(x,y)\right\}$$

where Ψ is a set of *n*_ψ_ equidistant orientations defined as $\Psi =\left\{\frac{2\pi }{n_{\psi }}i\;|\;0\leq \text{​}i\text{​}<\text{​}n_{\psi }\right\}$.

### 2.6. Tolerance to scaling

Tolerance to scaling is achieved in a similar way. Using the set *S*_S_ that defines the concerned *S*-COSFIRE filter, we form a new set *T*_υ_(*S*_S_) that defines a new filter, which is selective for a version of the prototype shape S that is scaled in size by a factor υ:

(5) $$T_{\upsilon }(S_{\text{S}})\overset{\text{def}}{=}\left\{(T_{\upsilon }(V_{f_{j_{i}}}),\upsilon \rho_{i},\phi_{i})\;|\;\forall \;(V_{f_{j_{i}}},\rho_{i},\phi_{i})\text{ ​}\in \text{ ​}S_{\text{S}}\right\}$$

For each tuple (*V*_*f*_*j*_*i*___, ρ_*i*_, ϕ_*i*_) in the original *S*-COSFIRE filter *S*_S_ that describes a certain vertex of the prototype shape, we provide a counterpart tuple (*T*_υ_(*V*_*f*_*j*_*i*___), υρ_*i*_, ϕ_*i*_) in the new set *T*_υ_(*S*_S_). The set *T*_υ_(*V*_*f*_*j*_*i*___) defines^1^ a V-COSFIRE filter that responds to a version of the vertex *f*_*j*_*i*__ scaled by the factor υ. The size of the concerned vertex and its distance to the center of the filter are scaled by the factor υ relative to the original values of the corresponding parameters.

A scale-invariant response is achieved by taking the maximum value of the responses of filters that are obtained with different values of the parameter υ:

(6) $${\tilde{r}}_{S_{\text{S}}}(x,y)\overset{\text{def}}{=}\underset{{}_{\upsilon \in ϒ}}{\max}\{r_{T_{\upsilon }(S_{\text{S}})}(x,y)\}$$

where ϒ is a set of υ values equidistant on a logarithmic scale defined as $ϒ=\{2^{\frac{i}{2}}\;|\;i\in \mathbb{Z}\}$.

### 2.7. Reflection invariance

As to reflection invariance we first form a new set *Ś*_S_ from the set *S*_S_ as follows:

(7) $${\overset{´}{S}}_{\text{S}}\overset{\text{def}}{=}\{({\overset{´}{V}}_{f_{j_{i}}},\rho_{i},\pi \text{​}-\text{​}\phi_{i})\;|\;\forall \;(V_{f_{j_{i}}},\rho_{i},\phi_{i})\in S_{\text{S}}\}$$

The set ${\overset{´}{V}}_{f_{j_{i}}}$ defines^1^ a new *V*-COSFIRE filter that is selective for the corresponding vertex *f*_*j*_*i*__ reflected about the *y*–axis. Similarly, the new *S*-COSFIRE filter *Ś*_S_ is selective for a reflected version of the prototype shape S also about the *y*−axis. A reflection-invariant response is achieved by taking the maximum value of the responses of the filters *S*_S_ and *Ś*_S_:

(8) $${\overset{´}{r}}_{S_{\text{S}}}(x,y)\overset{\text{def}}{=}\max\{r_{S_{\text{S}}}(x,y),r_{{\overset{´}{S}}_{\text{S}}}(x,y)\}$$

### 2.8. Combined tolerance to rotation, scaling, and reflection

An *S*-COSFIRE filter achieves tolerance to all the above geometric transformations by taking the maximum value of the rotation- and scale-tolerant responses of the filters *S*_S_ and *Ś*_S_ that are obtained with different values of the parameters ψ and υ:

(9) $${\bar{r}}_{S_{\text{S}}}(x,y)\overset{\text{def}}{=}\underset{{}_{\psi \in \Psi ,\upsilon \in ϒ}}{\max}\left\{{\hat{r}}_{{ℜ}_{\psi }(T_{\upsilon }(S_{\text{S}}))}(x,y),{\hat{r}}_{{ℜ}_{\psi }(T_{\upsilon }({\overset{´}{S}}_{\text{S}}))}(x,y)\right\}$$

## 3. Applications

In the following we demonstrate the effectiveness of the proposed *S*-COSFIRE filters by applying them in two practical applications: the spotting of keywords in handwritten manuscripts and the spotting of objects in complex scenes for the computer vision system of a domestic robot.

### 3.1. Spotting keywords in handwritten manuscripts

The automatic recognition of keywords in handwritten manuscripts is an application that has been extensively investigated for several decades (Plamondon and Srihari, 2000; Frinken et al., 2012). Despite this effort the problem has not been solved yet.

**Figure 5:**
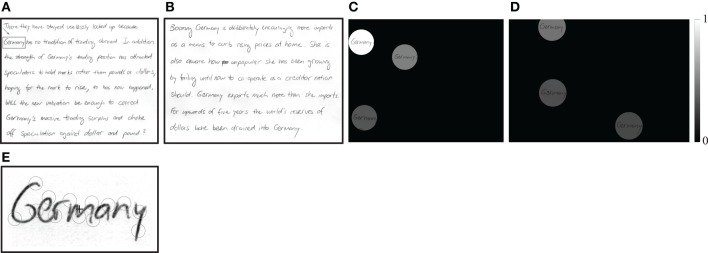
**An example of spotting the keyword “Germany” in (A,B) two handwritten manuscripts taken from the IAM offline database (Marti and Bunke, 2002)**. The indicated keyword “Germany” in **(A)** is used as a prototype to configure an *S*-COSFIRE filter. **(E)** The circles indicate the support areas of 13 *V*-COSFIRE filters that are used to provide input to the concerned *S*-COSFIRE filter with the “+” marker indicating its support center. **(C,D)** Normalized responses of this filter to the images in **(A,B)** rendered by shading of the spotted words. All six instances are detected. The strongest response is achieved for the word that was used for the configuration of the *S*-COSFIRE filter.

As a demonstration, in Figure 5 we show how to detect the keyword “Germany” in two handwritten manuscripts. We use the keyword prototype “Germany” that is shown enframed in Figure 5A to configure an *S*-COSFIRE filter that receives input from 13 *V*-COSFIRE filters, Figure 5E. Figures 5C,D show the responses of the concerned *S*-COSFIRE filter (*t*_1_ = 0.1, *t*_2_ = 0.75, *t*_3_ = 0.1, σ_0_ = 0.67, and α = 0.1.) to the two manuscript images^4^ in Figures 5A,B. It spots all the six instances of the keyword “Germany” and does not produce any false positives.

The *S*-COSFIRE filters that are selective for specific words may correspond to neurons or networks of neurons in a certain area in the posterior lateral-occipital cortex. This area receives input from V4 and is selective for combinations of vertices. It has been shown to play a role in the recognition of words and has been named Visual Word Form Area (Szwed et al., 2011).

### 3.2. Vision for a home tidying pickup robot

Daily service robots that perform routine tasks are becoming popular as household appliances. Such tedious tasks include, but are not limited to, vacuum cleaning, setting up and cleaning up a dinner table, tidying up toys, and organizing closets. The design of domestic robots is a growing research area (Bandera et al., 2012; Jiang et al., 2012).

**Figure 6:**
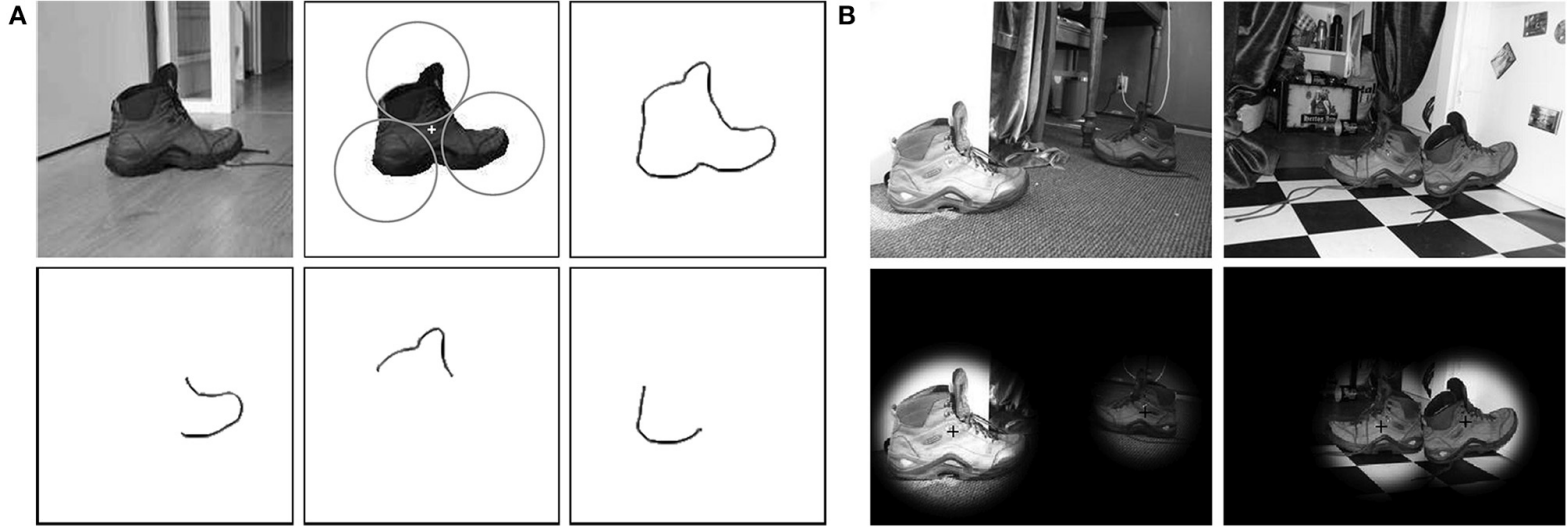
**Detection of shoes in complex scenes**. **(A)** A protoype shoe used for the configuration of an *S*-COSFIRE filter. The circles represent the non-overlapping supports of three *V*-COSFIRE filters, and the “+” marker indicates the center of support of the concerned *S*-COSFIRE filter. (Top right) The superimposed (inverted) thresholded responses (*t*_1_ = 0.3) of a bank of Gabor energy filters with one wavelength (λ = 4) and 16 orientations in intervals of π/8. (Bottom) Reconstructions of the local patterns for which the three resulting *V*-COSFIRE filters are selective. **(B)** Detection results to two input images (of size 256 × 342 pixels) from the RUG-Shoes data set with filenames (a) Shoes03_1.jpg, (c) Shoes17_2.jpg, (e) Shoes58_2.jpg, and (g) Shoes38_1.jpg.

We demonstrate how the *S*-COSFIRE filters that we propose can be used by a personal robot to visually recognize objects of interest in indoor environments. As an illustration we consider a task for a tidying pickup robot to detect shoes in different rooms of a home that match the prototype shoe shown in Figure 6A.

We use a segmented prototype image of the shoe to configure an *S*-COSFIRE filter. The concerned *S*-COSFIRE filter receives input from three *V*-COSFIRE filters that are selective for different parts of the shoe. These parts are automatically chosen by the system from a circular local neighborhood of a point of interest that is indicated by a “+” marker. In practice, the concerned point of interest and the radius of the corresponding local neighborhood are manually specified by the user. The radii of the three circles are automatically computed in such a way that the circles touch each other. For the configuration of the concerned *V*-COSFIRE filters we use a bank of Gabor energy filters^5^ with one wavelength (λ = 4) and 16 equidistant orientations $\left(\theta =\{\frac{\pi }{8}i\;|\;0\ldots 15\}\right)$, and we threshold the responses with *t*_1_ = 0.3. Within each of the three circles, we consider a number of concentric circles, the radii of which increment in intervals of 4 pixels starting from 0. For the concerned three *V*-COSFIRE filters as well as the *S*-COSFIRE filter we use the same values of parameters α (α = 0.67) and σ_0_ (σ_0_ = 0.1) in order to allow the same tolerance in the position of the involved edges and curvatures.

We created a data set that we call RUG-Shoes of 60 color images (of size 256 × 342 pixels) by taking pictures in different rooms of the same house. Of these images, 39 contain a pair of shoes of interest, another nine contain a single shoe and the remaining 12 do not contain any shoes. The distance above ground of the digital camera was varied between 50 cm and 1 m. All pictures of shoes were taken from the side view of the corresponding shoes. The shoes were, however, arranged in different orientations and their distances from the camera varied by at most 25% as compared to the distance which we used to take the image of the prototype shoe. We made the RUG-Shoes data set publicly available^6^.

We use the configured *S*-COSFIRE filter to detect shoes in the data set of 60 images. We first convert every color image to grayscale and subsequently apply the concerned *S*-COSFIRE filter in reflection-, scale- $\left(\upsilon \in \left\{\frac{3}{4},1,\frac{5}{4}\right\}\right)$ and partially rotation-invariant $\left(\psi \text{​}\in \text{​}\left\{-\frac{\pi }{8},0,\frac{\pi }{8}\right\}\right)$ mode. The Gabor energy filters that we use to provide inputs to the *V*-COSFIRE filters are applied with isotropic suppression (Grigorescu et al., 2004) in order to reduce responses to texture. We threshold the responses of the concerned *S*-COSFIRE filter with *t*_3_ = 0.1 and for each image we consider only the highest two responses. We obtain a perfect detection and recognition performance for all the 60 images in the RUG-Shoes data set. This means that we detect all the shoes in the given images with no false positives. Figure 6B illustrates the detection of some shoes in two of the images.

## 4. Discussion

The trainable *S*-COSFIRE filters that we propose are part of a hierarchical object recognition approach that shares similarity with the ventral stream of visual cortex. In the first layer we detect lines and edges by Gabor filters, which are inspired by the function of orientation-selective cells in primary visual cortex (Daugman, 1985). Their responses are projected to a second layer and used by *V*-COSFIRE filters that detect vertices and curved contour segments. In our previous work (Azzopardi and Petkov, 2013b), we showed that such filters give responses that are qualitatively similar to a class of cells in area V4 in visual cortex. Finally, in a third layer we have *S*-COSFIRE filters that combine the responses of certain *V*-COSFIRE filters. Such a filter is selective for a given spatial configuration of vertices and curved contour segments that defines a simple to moderately complex shape. *S*-COSFIRE filters share similar properties with shape-selective neurons in inferotemporal cortex, which provided inspiration for this work.

This hierarchical object recognition approach is, however, not restricted to three layers. The addition of further layers may be more appropriate for prototype objects of higher deformation complexity. For instance, let us consider a prototype shape of a simplistic human-body figure that is composed of a head, a pair of eyes, a nose, a mouth, two arms, two hands, a torso, two legs, and two feet. We may configure an *S*-COSFIRE filter to be selective for the entire body with its center being at the center of mass of the body. Such a filter receives input from *V*-COSFIRE filters that are selective for distinct body parts. With this type of configuration the tolerance in the position of the body parts is computed with the same function that depends on the distance from the center of the *S*-COSFIRE filter. However, we know that certain body parts may require more tolerance or may be more correlated than others. For instance, the positions of the eyes, the nose and the mouth depend more on the position of the head than on the position of the legs. By taking this aspect in consideration it would be better to construct a hierarchical filter in the following way: configure an *S*-COSFIRE filter to be selective for the spatial arrangement of the head components (eyes, nose, and mouth), an *S*-COSFIRE filter for a hand and an arm, another one for a foot and a leg and a fourth one for the torso. Then, the responses of these four *S*-COSFIRE filters may be used as inputs to another, more complex *S*-COSFIRE filter.

The configuration of an *S*-COSFIRE filter determines which responses of which *V*-COSFIRE filters need to be multiplied in order to obtain the output of the filter. The number of *V*-COSFIRE filters used is a model parameter that is specified by the user. This value depends on the shape complexity of the concerned prototype (as represented by the number of vertex features). The selectivity of an *S*-COSFIRE filter increases with an increasing number of *V*-COSFIRE filters. The sizes of the *V*-COSFIRE supports and their position are automatically determined in such a way that they do not overlap each other. In future work, we will incorporate a learning mechanism in the configuration stage. It will use multiple prototype examples of the object of interest (instead of only one prototype that we use here) and negative examples (e.g., other objects and scenes). It will learn the optimal number of *V*-COSFIRE filters as well as the size and position of their support in order to maximize selectivity and generalization abilities.

An *S*-COSFIRE filter achieves a response when all parts of a shape of interest are present in a specific spatial arrangement around a given point in an image. The rigidity of this geometrical configuration may vary according to the application at hand. The standard deviation of a blurring (Gaussian) function that we use to allow for some tolerance depend on the distance from the center of the concerned *S*-COSFIRE filter: it grows linearly with a rate that is defined by the parameter α. Small values of α are more appropriate for the selectivity of rigid objects. Generalization ability increases with an increasing value of α. This mechanism is inspired by neurophysiological evidence that the average diameter of receptive fields of some neurons in visual cortex increases with the eccentricity (Gattass et al., 1988).

The specific type of function that we use to combine the responses of costituent (*V*-COSFIRE) filters for the considered applications is a weighted geometric mean. This output function, which is also used to compute a *V*-COSFIRE filter response, proved to give better results than various forms of addition. Furthermore, there is psychophysical evidence that human visual processing of shape is likely performed by a non-linear neural operation that multiplies afferent responses (Gheorghiu and Kingdom, 2009). In future work, we plan to experiment with functions other than (weighted) geometric mean.

The application of the home tidying robot in section 3.2 demonstrates the benefits of the rotation, scale and reflection invariances that we use. With one *S*-COSFIRE filter that is configured by a single prototype, the filter is able to achieve responses to different views of the object used for training. While this ability implies more operations, the computational cost does not grow linearly with the number of considered views. This is attributable to the fact that the responses of the bank of Gabor filters at the bottom layer can be shared among the involved *V*-COSFIRE filters, irrespective of the view. We refer the reader to Azzopardi and Petkov (2013a,b) for the technical details. The majority of the new operations required due to the invariances are shifting computations, which have very low computational cost. In practice, the shoe-selective filter used in section 3.2 takes 3.5 s to process an image (256 × 342 pixels) with no invariances, and less than 5 s with rotation-, scale-, and reflection-invariance.

The proposed *S*-COSFIRE filters are particularly useful due to their versatility and selectivity, in that an *S*-COSFIRE filter can be configured to be selective for any given deformable object and used to detect other objects embedded in complex scenes that are perceptually similar to it. This effectiveness is attributable to taking into account the mutual spatial positions of the responses of certain *V*-COSFIRE filters that are selective for simpler object parts.

## 5. Conclusions

The *S*-COSFIRE filters that we propose are highly effective to detect and recognize deformable objects that are embedded in complex scenes without prior segmentation. This effectiveness is due to the deployment of both the presence of certain object-characteristic features and their mutual spatial arrangement. They are versatile shape detectors as they can be trained to be selective for any given visual pattern of interest.

An *S*-COSFIRE filter is conceptually simple and easy to implement: the filter output is computed as the weighted geometric mean of blurred and shifted responses of simpler *V*-COSFIRE filters.

### Conflict of interest statement

The authors declare that the research was conducted in the absence of any commercial or financial relationships that could be construed as a potential conflict of interest.

## Footnotes

^1^The Matlab implementation of a *V*-COSFIRE filter can be downloaded from http://matlabserver.cs.rug.nl/

^2^Here we use a bank of Gabor filters with five wavelengths λ = {4, 4$\sqrt{2}$, 8, 8$\sqrt{2}$, 16} and six equidistant orientations $\theta \in \left\{0,\frac{\pi }{6},\frac{\pi }{3},\frac{\pi }{2},\frac{2\pi }{3},\frac{5\pi }{6}\right\}$

^3^We refer to Azzopardi and Petkov (2013b) for the technical details about the invariance that is achieved by a *V*-COSFIRE filter.

^4^The images in Figures 5A,B are extracted from the files named b01-049.png and b01-044.png, respectively, in the IAM offline database.

^5^The response of a Gabor energy filter is computed as the L2-norm of the responses of a symmetric and anti-symmetric Gabor filters.

^6^The RUG-Shoes data set can be downloaded from http://matlabserver.cs.rug.nl/

## References

[B1] Almazan J. Fornes A. Valveny E. (2012). A non-rigid appearance model for shape description and recognition. Pattern Recogn. 45, 3105–3113 10.1016/j.patcog.2012.01.010

[B2] Azzopardi G. Petkov N. (2012). A CORF computational model of a simple cell that relies on LGN input outperforms the Gabor function model. Biol. Cybernet. 106, 177–189 10.1007/s00422-012-0486-6 22526357

[B3] Azzopardi G. Petkov N. (2013a). Automatic detection of vascular bifurcations in segmented retinal images using trainable COSFIRE filters. Pattern Recogn. Lett. 34, 922–933 10.1016/j.patrec.2012.11.002

[B4] Azzopardi G. Petkov N. (2013b). Trainable COSFIRE Filters for Keypoint Detection and Pattern Recognition. IEEE Trans. Pattern Anal. Mach. Intell. 35, 490–503 10.1109/TPAMI.2012.106 22585100

[B5] Azzopardi G. Rodriguez Sanchez A. Piater J. Petkov N. (2014). A push-pull CORF model of a simple cell with antiphase inhibition improves SNR and contour detection. PLoS ONE 9:e98424 10.1371/journal.pone.0098424 25057813 PMC4109930

[B6] Bandera J. P. Rodriguez J. A. Molina-Tanco L. Bandera A. (2012). A survey of vision-based architectures for robot learning by imitation. Int. J. Human. Robot. 9:1250006 10.1142/S0219843612500065

[B7] Belongie S. Malik J. Puzicha J. (2002). Shape matching and object recognition using shape contexts. IEEE Trans. Pattern Anal. Mach. Intell. 24, 509–522 10.1109/34.993558 16285381

[B8] Brincat S. L. Connor C. E. (2004). Underlying principles of visual shape selectivity in posterior inferotemporal cortex. Nat. Neurosci. 7, 880–886 10.1038/nn1278 15235606

[B9] Daugman J. G. (1985). Uncertainty relation for resolution in space, spatial-frequency, and orientation optimized by two-dimensional visual cortical filters. J. Optic. Soc. Am. Optic. Image Sci. Vis. 2, 1160–1169 10.1364/JOSAA.2.001160 4020513

[B10] DiCarlo J. Cox D. (2007). Untangling invariant object recognition. Trends Cogn. Sci. 11, 333–341 10.1016/j.tics.2007.06.010 17631409

[B11] Edelman S. Intrator N. (2003). Towards structural systematicity in distributed, statically bound visual representations. Cogn. Sci. 27, 73–109 10.1207/s15516709cog2701_3

[B12] Fei-Fei L. Fergus R. Perona P. (2007). Learning generative visual models from few training examples: an incremental Bayesian approach tested on 101 object categories. Comput. Vis. Image Understand. 106, 59–70 2nd International Workshop on Generative-Model Based Vision, Washington, DC, 2005. 10.1016/j.cviu.2005.09.01216566508

[B13] Fergus R. Perona P. Zisserman A. (2003). Object class recognition by unsupervised scale-invariant learning, in Proceedings of the 2003 IEEE Computer Society Conference on Computer Vision and Pattern Recognition, Vol. 2, Technical Committee on Pattern Analysis and Machine Intelligence (TCPAMI) (Madison, WI), 264–271

[B14] Fidler S. Boben M. Leonardis A. (2008). Similarity-based cross-layered hierarchical representation for object categorization, in 2008 IEEE Conference on Computer Vision and Pattern Recognition, Vol. 1–12 (Anchorage, AK), 525–532 10.1109/CVPR.2008.4587409

[B15] Fidler S. Leonardis A. (2007). Towards scalable representations of object categories: learning a hierarchy of parts, in 2007 IEEE Conference on Computer Vision and Pattern Recognition, Vol. 1–8 (Minneapolis, MN), 2295–2302 10.1109/CVPR.2007.383269

[B16] Frinken V. Fischer A. Manmatha R. Bunke H. (2012). A novel word spotting method based on recurrent neural networks. IEEE Trans. Pattern Anal. Mach. Intell. 34, 211–224 10.1109/TPAMI.2011.113 21646681

[B17] Gattass R. Sousa A. P. Gross C. G. (1988). Visuotopic organization and extent of v3 and v4 of the macaque. J. Neurosci. 8, 1831–1845 3385477 10.1523/JNEUROSCI.08-06-01831.1988 PMC6569322

[B18] Gheorghiu E. Kingdom F. A. A. (2009). Multiplication in curvature processing. J. Vis. 9, 1–17 10.1167/9.2.23 19271933

[B19] Ghosh A. Petkov N. (2005). Robustness of shape descriptors to incomplete contour representations. IEEE Trans. Pattern Anal. Mach. Intell. 27, 1793–1804 10.1109/TPAMI.2005.225 16285377

[B20] Goh W. B. (2008). Strategies for shape matching using skeletons. Comput. Vis. Image Understand. 110, 326–345 10.1016/j.cviu.2007.09.01317354727

[B21] Grigorescu C. Petkov N. (2003). Distance sets for shape filters and shape recognition. IEEE Trans. Image Process. 12, 1274–1286 10.1109/TIP.2003.816010 18237892

[B22] Grigorescu C. Petkov N. Westenberg M. A. (2004). Contour and boundary detection improved by surround suppression of texture edges. Image Vis. Comput. 22, 609–622 10.1016/j.imavis.2003.12.004

[B23] Jiang Y. Lim M. Zheng C. Saxena A. (2012). Learning to place new objects in a scene. Int. J. Robot. Res. 31, 1021–1043 10.1177/027836491243878115687795

[B24] Kobatake E. Tanaka K. (1994). Neuronal selectivities to complex object features in the ventral visual pathway of the macaque cerebral-cortex. J. Neurophysiol. 71, 856–867 8201425 10.1152/jn.1994.71.3.856

[B25] Latecki L. Lakaemper R. Wolter D. (2005). Optimal partial shape similarity. Image Vis. Comput. 23, 227–236 11th International Conference on Discrete Geometry for Computer Imagery, Italian Inst Philosoph Studies, Naples, Italy, Nov 19-21, 2003. 10.1016/j.imavis.2004.06.01520163981

[B26] Lauer F. Suen C. Y. Bloch G. (2007). A trainable feature extractor for handwritten digit recognition. Pattern Recogn. 40, 1816–1824 10.1016/j.patcog.2006.10.011

[B27] Ling H. Jacobs D. W. (2007). Shape classification using the inner-distance. IEEE Trans. Pattern Anal. Mach. Intell. 29, 286–299 10.1109/TPAMI.2007.41 17170481

[B28] Marr D. (1982). Vision: A Computational Investigation Into The Human Respresentation and Processing of Visual Information. New York, NY: Freeman

[B29] Marr D. Nishihara H. K. (1978). Representation and recognition of spatial-organization of 3-dimensional shapes. Proc. R. Soc. London B Biol. Sci. 200, 269–294 10.1098/rspb.1978.0020 24223

[B30] Marti U.-V. Bunke H. (2002). The IAM-database: an english sentence database for offline handwriting recognition. Int. J. Doc. Anal. Recogn. 5, 39–46 10.1007/s100320200071

[B31] Mel B. W. Fiser J. (2000). Minimizing binding errors using learned conjunctive features (vol 12, pg 247, 1999). Neural Comput. 12, 731–762 10.1162/089976600300015574 10770830

[B32] Plamondon R. Srihari S. (2000). On-line and off-line handwriting recognition: a comprehensive survey. IEEE Trans. Pattern Anal. Mach. Intell. 22, 63–84 10.1109/34.824821

[B33] Riesenhuber M. Poggio T. (1999). Hierarchical models of object recognition in cortex. Nat. Neurosci. 2, 1019–1025 10.1038/14819 10526343

[B34] Rodríguez-Sánchez A. J. Tsotsos J. K. (2012). The roles of endstopped and curvature tuned computations in a hierarchical representation of 2d shape. PLoS ONE 7:e42058 10.1371/journal.pone.0042058 22912683 PMC3415424

[B35] Roelfsema P. R. (2006). Cortical algorithms for perceptual grouping. Annu. Rev. Neurosci. 29, 203–227 10.1146/annurev.neuro.29.051605.112939 16776584

[B36] Scalzo F. Piater J. (2005). Statistical learning of visual feature hierarchies, in IEEE Computer Society Conference on Computer Vision and Pattern Recognition - Workshops, 2005. CVPR Workshops (San Diego, CA), 44

[B37] Serre T. Wolf L. Bileschi S. Riesenhuber M. Poggio T. (2007). Robust object recognition with cortex-like mechanisms. IEEE Trans. Pattern Anal. Mach. Intell. 29, 411–426 10.1109/TPAMI.2007.56 17224612

[B38] Szwed M. Dehaene S. Kleinschmidt A. Eger E. Valabregue R. Amadon A. (2011). Specialization for written words over objects in the visual cortex. Neuroimage 56, 330–344 10.1016/j.neuroimage.2011.01.073 21296170

[B39] Tsotsos J. (1990). Analyzing vision at the complexity level. Behav. Brain Sci. 13, 423–444 10.1017/S0140525X0007957719624853

